# MorusDB: a resource for mulberry genomics and genome biology

**DOI:** 10.1093/database/bau054

**Published:** 2014-06-11

**Authors:** Tian Li, Xiwu Qi, Qiwei Zeng, Zhonghuai Xiang, Ningjia He

**Affiliations:** State Key Laboratory of Silkworm Genome Biology, Southwest University, Chongqing 400715, China

## Abstract

Mulberry is an important cultivated plant that has received the attention of biologists interested in sericulture and plant–insect interaction. *Morus notabilis*, a wild mulberry species with a minimal chromosome number is an ideal material for whole-genome sequencing and assembly. The genome and transcriptome of *M. notabilis* were sequenced and analyzed. In this article, a web-based and open-access database, the Morus Genome Database (MorusDB), was developed to enable easy-to-access and data mining. The MorusDB provides an integrated data source and an easy accession of mulberry large-scale genomic sequencing and assembly, predicted genes and functional annotations, expressed sequence tags (ESTs), transposable elements (TEs), Gene Ontology (GO) terms, horizontal gene transfers between mulberry and silkworm and ortholog and paralog groups. Transcriptome sequencing data for *M. notabilis* root, leaf, bark, winter bud and male flower can also be searched and downloaded. Furthermore, MorusDB provides an analytical workbench with some built-in tools and pipelines, such as BLAST, Search GO, Mulberry GO and Mulberry GBrowse, to facilitate genomic studies and comparative genomics. The MorusDB provides important genomic resources for scientists working with mulberry and other Moraceae species, which include many important fruit crops. Designed as a basic platform and accompanied by the SilkDB, MorusDB strives to be a comprehensive platform for the silkworm–mulberry interaction studies.

**Database URL**: http://morus.swu.edu.cn/morusdb.

## Introduction

*Morus* (mulberry) is a genus of flowering plants from the family Moraceae. The deciduous mulberry tree is an economically important food crop for the domesticated silkworm. Human beings have used the mulberry–silkworm interaction for at least 5000 years in the sericulture industry, which greatly changed world history. More than 150 mulberry species were registered, among which *Morus notabilis* C.K.Schneid was first recorded by Schneide in 1916. It became well known as a naturally available mulberry species, which had 14 chromosomes according to the cytological and morphological studies (1). *Morus* *notabilis* was discovered in Sichuan province, Southwest of China, and is named Chuansang, has seven chromosomal pairs as determined by cytological studies (2). Chuansang is a dioecious tree. It is 9–15 m tall and has grayish brown bark, orbicular leaves with triangular serrated and paired male flower, which is 4–5 cm long. As Chuansang has a minimal chromosomal number, it was selected for genome sequencing in the *Morus* Genome Project (MGP). The MGP was performed on Illumina Hiseq 2000 platform using a whole-genome shotgun sequencing strategy. Twelve libraries were constructed and sequenced to produce 78.34 billion high-quality reads (236-fold genome coverage), from which we assembled a 330.79-Mb genome using SOAPdenovo 2 (3). The MGP also included transcriptomic sequencing of the Chuansang root, branch bark, winter bud, male flower and leaf and expressed sequence tag (EST) sequencing of a combined cDNA library from the five tissues mentioned above (2).

Combined with data of silkworm and other closely related plants, the high-throughput mulberry genomic data provide bases and references for researchers to carry out studies on mulberry genomics, comparative genomics, biology and interactions between silkworm and mulberry. However, it is difficult to use this data efficiently without a platform and tools. Based on the MGP data, we constructed the MorusDB, a database and platform for researchers to search, analyze, collect and share the mulberry genomic and related data.

## Data sets and methods

### System implementation

**Figure 1.**
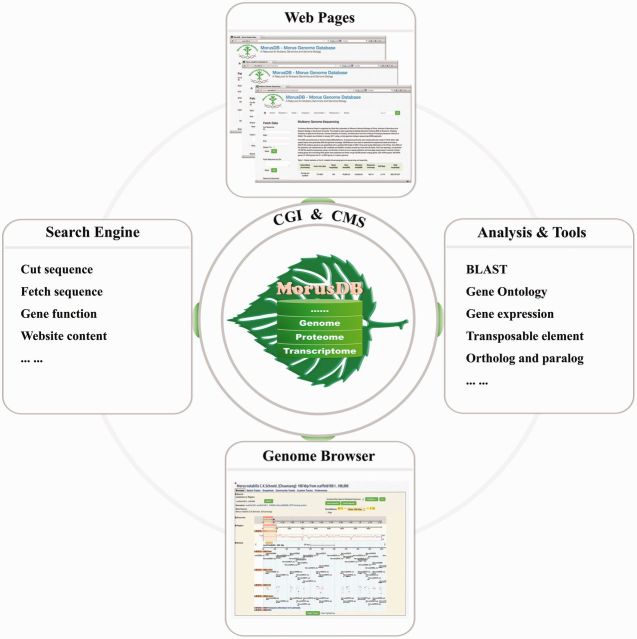
The framework of MorusDB. MorusDB core is implemented in MySQL database for storing data and contents. CGI programs and CMS were used to constitute the intermediate layer, which was used to carry out analysis and manage and display data and contents to the outer layer, web pages.

The server that MorusDB depends on was built with Linux Ubuntu Sever 12.04, Apache 2, MySQL Server 5.5 and PHP 5.3. The framework of MorusDB is composed of three layers (Figure 1). A relational database, morusdb, is the core layer and implemented in the MySQL relational database management system. All mulberry data and information were stored in MySQL tables so that they can be managed, searched and displayed efficiently. Common gateway interface (CGI) programs and content management system (CMS) constitute the intermediate layer. The CGIs were mainly developed using Perl, PHP, JavaScript and C programming languages, with which we developed scripts for fetching and cutting sequences, searching genes, performing analyses on gene expression, transposable elements and homologous genes, as well as tools and pipelines for aligning sequence and searching gene ontology. The front end is managed by the Drupal (https://drupal.org), an open source CMS that is distributed under the terms of the GNU's Not Unix (GNU) General Public License. Results of search and analyses will be submitted to the Drupal CMS and displayed to user end. The mulberry genome browser, Mulberry GBrowse, is driven by the Generic Genome Browser (4, 5), one of the Generic Model Organism Database (http://gmod.org) components for manipulating and displaying annotations on genomes. The Mulberry GBrowse was configured following instructions so that it can access mulberry data in the morusdb database.

### Data and processing

The *M.* *notabilis* genomic assembly is composed of 110 760 scaffolds, from which 27 085 protein-coding genes consisting of 124 550 exons, 81 rRNA genes, 560 tRNA genes, 311 snRNA genes, 233 microRNA genes and 324 736 repetitive sequences were predicted using *de* *novo* and homologous methods (Table 1). Translated proteins were aligned against the latest nonredundant protein database (nr) from the GenBank and UniProtKB/SwissProt from the UniProt release 4.0 (6) using BLASTP 2.2.26 (7, 8) under *E*-value threshold of 1e-5 to obtain functional references, which were then manually curated. Protein domains and Gene Ontologies (GO) were predicted by searching the SMART (9) and InterPro (10) databases using a local batch script and InterProScan5 (10), respectively. The repetitive elements include 53 725 classified transposable elements (TE) and 271 011 other repeats. The scaffolds accompanied by all the predicted genes, repeats and annotations were processed and imported into the morusdb. Meanwhile, the *M.* *notabilis* genome data have been deposited in National Center for Biotechnology Information (NCBI) under BioProject no. PRJNA202089 and GenBank no. ATGF00000000.1.

**Table 1.** Data set of mulberry *M. notabilis* deposited in MorusDB (1)

	No.	Size (base pairs / amino acids)
Genome		
Scaffold	110 760	330 791 087
Gene	27 085	77 647 891
CDS	124 550	29 437 365
Protein	27 085	9 785 370
rRNA	81	12 174
tRNA	560	43 692
snRNA	311	34 162
microRNA	233	26 520
Repeats		
Known transposable element	53 725	74 910 659
Others	271 011	53 073 173
Transcriptome		
Libraries for mRNA sequencing		
Leaf	25 853 672	2 326 830 480
Root	25 583 558	2 302 520 220
Branch bark	25 969 690	2 337 272 100
Winter bud	26 774 498	2 409 704 820
Male flower	25 688 148	2 311 933 320
cDNA library		
EST of above tissues	9573	4 595 661

To identify orthologous and paralogous groups (OPGs) of *M.* *notabilis* genes (MOPGs), the latest version of protein sequences of relative plants were downloaded. *Arabidopsis thaliana* proteins were downloaded from the FTP server (ftp://ftp.arabidopsis.org/home/tair/Proteins/TAIR10_protein_ lists) of TAIR database (http://www.ara bidopsis.org). Proteins of *Populus trichocarpa*, *Malus domestica* and *Fragaria vesca* were downloaded from the FTP server (ftp://ftp.jgi-psf.org/pub/compgen/phytozome/v9.0) of DOE JGI (http://www.jgi.doe.gov). Thereafter, the OPGs of the aforementioned five plants were predicted using OrthoMCL v2.0.9. Proteins were all-against-all aligned using BLASTP 2.2.26 (7, 8) under *E*-value cutoff of 1e-6. The OrthoMCL (11) pipeline was then run under the percentMatchCutoff of 50. Finally, 24 065 MOPGs, including 19 858 *M.* *notabilis* genes, were obtained from a collection of 231 832 proteins and imported into the morusdb.

The Illumina sequencing of *M. notabilis* transcriptome from the five tissues generated 11.7 Gb sequences, which were deposited in the NCBI SRA under accession number of SRP040752. The transcriptome sequences were mapped to the genome sequences using SOAPaligner 2.20 (http://soap.genomics.org.cn/soapaligner.html) to determine gene expression levels using number of reads per kilobase per million mapped reads (RPKM) (12). Features of gene expression in the five tissues were then analyzed based on the RPKMs. In addition to screen out the tissue-specific expressed genes and housekeeping genes (2), we also found 8814 genes that encoded two or more isoforms and 4.97 Mb of untranslated region (UTR) from 16 513 mulberry genes. Meanwhile, the 9573 ESTs sequenced from the combined cDNA library of the five tissues were aligned against the scaffolds using BLAT (13).

## Results

**Figure 2.**
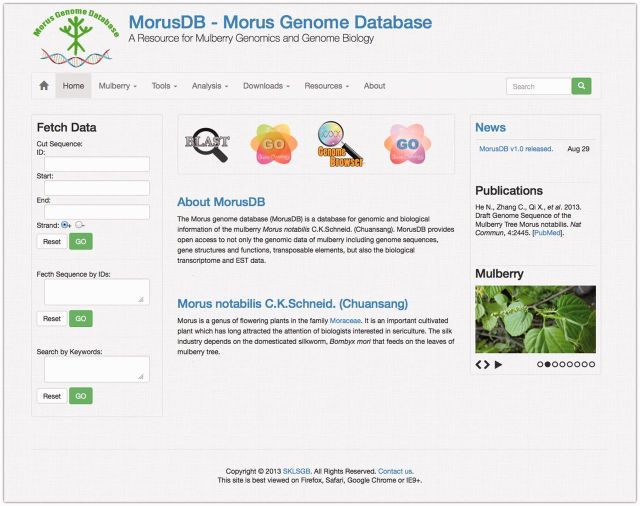
A snapshot of the MorusDB home page. The home page describes the mulberry genome sequencing project and provides links to all other parts of the website.

We intended to provide users with an efficient and direct way to access MorusDB data. Therefore, a clean and simple home page (Figure 2) was built to enable users to search for the mulberry data and information, perform analyses and download all data just by clicking hyperlinks on a navigation menu on top of the page. MorusDB is also a responsive Web site and friendly to both mobile and desktop devices.

### Search database

**Figure 3.**
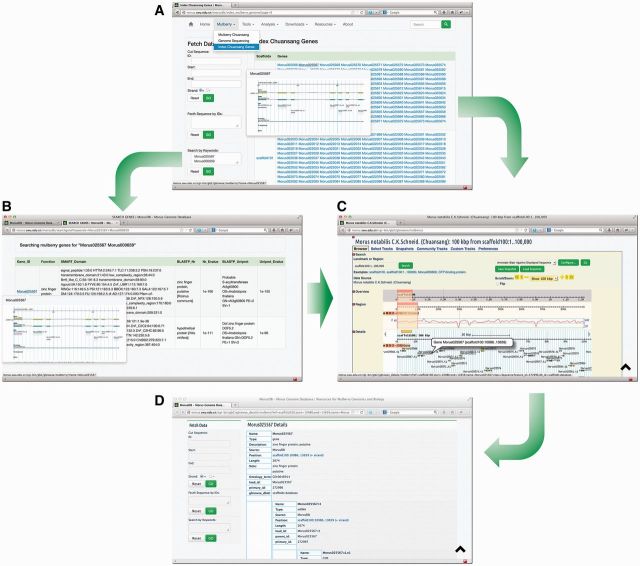
Searching and browsing *M. notabilis* genes. Genes of *M. notabilis* can be browsed or searched (**A**) using tools ‘Index of Chuansang Genes’ and ‘Search by Keywords’. Gene structures can be bird-eye viewed by mouse-hovering gene IDs (A and **B**). Users can click on these IDs to open the Mulberry GBrowse to see the detailed information (**C** and **D**).

MorusDB contents, including mulberry knowledge, news, tools, etc., can be quickly searched with top-right search engine. Using Fetch Data tool on the left side of pages, users can easily cut a sequence region, retrieve sequences in batches and search genes with keywords (Figure 3). Homologous sequence search can be carried out using the NCBI-BLAST 2.2.26 (7, 8) and the faster and more sensitive AB-BLAST 3.0 (http://blast.advbiocomp.com) against genome data of mulberry, silkworm and the public nr and UniProt databases. The search results can be printed out as a standard output with alignment figures or parsed into a tabular format under a given number of best matches and best hits.

### Mulberry GO

**Figure 4.**
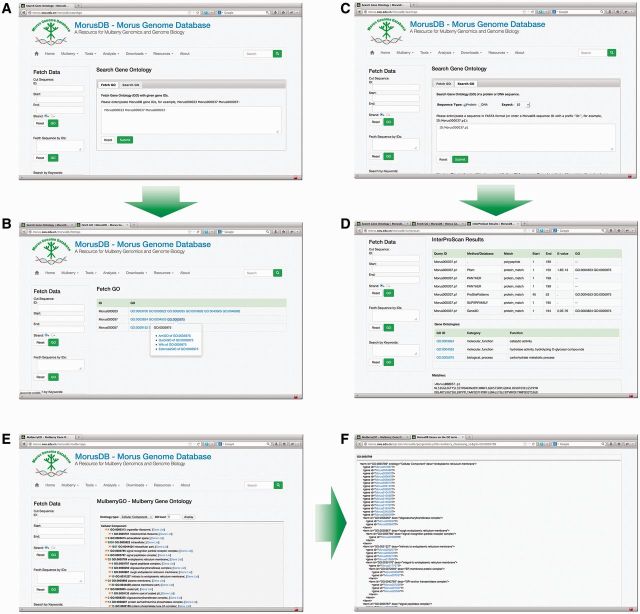
Snapshots of searching *M. notabilis* gene ontologies (GOs). Researchers can fetch GOs by gene IDs using Fetch GO (**A** and **B**) and search GOs by sequences or sequence IDs using Search GO (**C** and **D**). All *M. notabilis* GOs can also be birds-eye viewed with Browse GO (**E** and **F**).

*Morus notabilis* gene ontologies can be analyzed using the Mulberry GO (Figure 4) tool package. GO can be retrieved using Fetch GO with given gene IDs and searched using Search GO, which will align given sequence against the InterPro database by running the InterProScan 5 locally. Both tools will list GO IDs with explanation hyperlinks to AmiGO (14), QuickGO (15), GONUTS (16) and External2GO (17). Browse GO provides a birds-eye view of the *M.* *notabilis* GO. The Browse GO classified *M.* *notabilis* genes into three GO categories, cellular component, molecular function and biological process. *Morus* *notabilis* genes under each subcategory were listed and linked to detailed gene information pages.

### Mulberry Transcriptome, TEs and MOPGs

**Figure 5.**
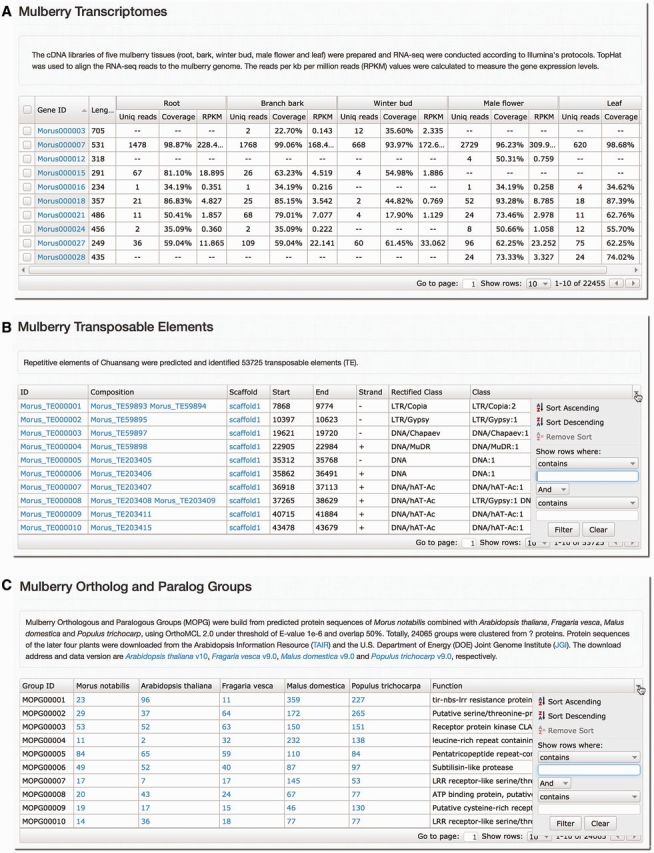
Screenshots of analyses on *M. notabilis* transcriptome, TEs and OPGs. Gene expressions in the five tissues (**A**), TEs (**B**) and OPGs (**C**) of the *M. notabilis* can be searched and filtered by keyword or number in each column of the tables.

To manage large data efficiently, *M.* *notabilis* gene expression patterns in the five tissues, classified transposable elements and orthologous and paralogous groups were stored in MySQL tables, which can be exported to the JavaScript Object Notation (JSON, http://www.json.org) data. The JSON data can be easily sorted, filtered and exported using the jqxGrid (http://www.jqwidgets.com), which is a powerful datagrid widget built entirely with open web standards and offers rich functionality. Therefore, researchers can search each column by keywords or numbers (Figure 5). Hyperlinks were added to IDs and numbers for a quick accession of detailed information and sequence.

### Mulberry GBrowse

The Mulberry GBrowse is a comprehensive and powerful analysis tool, which has integrated the *M.* *notabilis* genes, CDSs, proteins and TEs, as well as the transcriptome data. Using this tool, users can easily browse and search *M.* *notabilis* data on a large scale and with a graphic interface, and conveniently view and fetch detailed gene information including location, annotation, GO, sequences, etc. (Figure 3).

## Conclusion and future perspective

The mulberry genomic information provides a foundation for many kinds of studies: revealing the global organization of the mulberry genome, enabling studies of comparative genomics among mulberry and other eudicot species, accelerating gene identification and characterization and applying ‘omics’ technologies to better understand the biological phenomena of mulberry. The MorusDB is a comprehensive resource and platform that provides researchers with not only the present mulberry genome data but also tools for carrying out data analysis. Mulberry and silkworm were both sequenced as a plant–herbivore pair so that they are ideal objects and materials for interactional studies. In this regard, the MorusDB and SilkDB (18, 19), both maintained in our laboratory, not only provide kinds of data, but also form an associated platform for related studies in the future.

Beyond its initial release, MorusDB is a continuous effort to follow advances in studies on mulberry and expand, revise and improve the mulberry data of genome, transcriptome and proteome, as well as biological information. Furthermore, we will keep making efforts to develop MorusDB built-in tools and pipelines to facilitate and promote studies on mulberry.
